# Breast Abscessed Cancer in Nonlactating Women in Tropical Environment: Radiological, Bacteriological, and Anatomopathological Features about 3 Cases

**DOI:** 10.1155/2017/1639847

**Published:** 2017-08-27

**Authors:** Mazamaesso Tchaou, Tchin Darre, Ayi Kossi Amavi, Kokou Kouliwa Kanassoua, Bidamin N'Timon, Lantam Sonhaye, Lama-Kegdigoma Agoda-Koussema, Komlavi Adjenou

**Affiliations:** ^1^University Teaching Hospital of Lomé, Department of Radiology, Lomé, Togo; ^2^University Teaching Hospital of Lomé, Department of Pathology, Lomé, Togo; ^3^University Teaching Hospital of Lomé, Department of General Surgery, Lomé, Togo

## Abstract

The association of breast cancer and abscess is rare in daily practice. The authors report a short series of 3 cases of cancer of the breast in nonlactating women presented as breast abscess, reviewing aspects in radiology (ultrasound and mammography), correlating them with the histopathology findings and the bacteriological profile of the isolated germs.

## 1. Introduction

Inflammatory and infectious breast mastitis, including breast abscesses, are generally benign diseases that rarely harbour malignancy. It occurs most frequently in pregnancy and lactation period but may occur outside of pregnancy and lactation [1]. Although the occurrence of cancer is not anticipated in true breast abscesses, it can occasionally happen.

In this paper, we present a short series of 3 cases of cancer of the breast in nonlactating women presented as breast abscess, reviewing aspects in radiology (ultrasound and mammography), correlating them with the histopathology and the bacteriological profile of the isolated germs.

## 2. Case Presentation

### 2.1. Case 1

**Figure 1:**
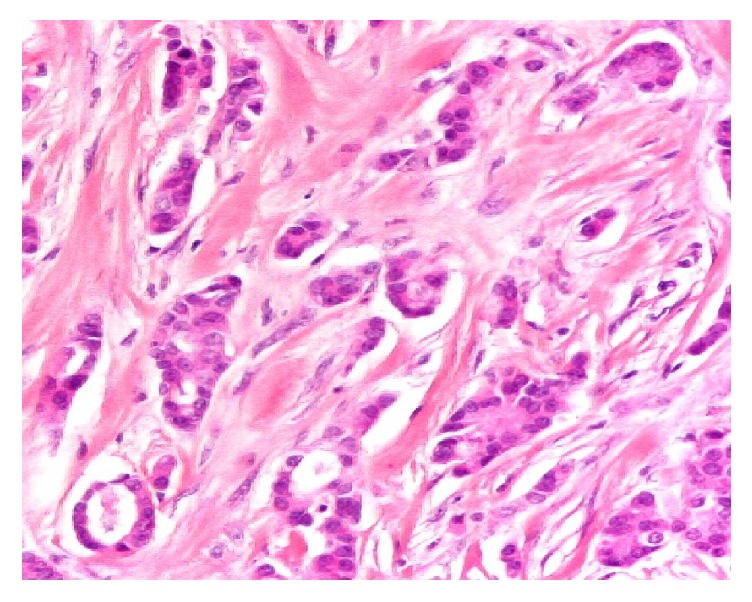
Histologic image of infiltrative carcinoma with subacute mastitis. G ×100, HE* (Collection of the Department of Pathology, University Teaching Hospital of Lomé)*.

A 31-year-old nonlactating and nonpregnant woman presented with a painful palpable mass in her left breast for six weeks. She was initially self-treated by automedication with traditional medicine without any improvement. At the hospital, she subsequently underwent a mammogram and an ultrasound. Mammogram demonstrated a retroareolar overdensity of the left breast, with undefined and irregular posterior limits, in a dense breast. Ultrasound noted a heterogeneous retroareolar mass of 5 cm of the left breast associated with dilatation of the galactophoric ducts contenting finely echoic fluid, with homolateral multiple enlarged axillary lymph nodes. The mass was diagnosed as an abscess and a fine needle puncture showed the pus of which bacteriologic exam had isolated a germ (*Staphylococcus aureus*) sensitive to penicillin. A correct antibiotic treatment was conducted without any improvement on ultrasound controls. The patient subsequently underwent ultrasound-guided core needle biopsy of the left breast mass. Histopathologic exam of the biopsy specimen showed an infiltrative canalar carcinoma with subacute mastitis (Figure 1).

### 2.2. Case 2

**Figure 2:**
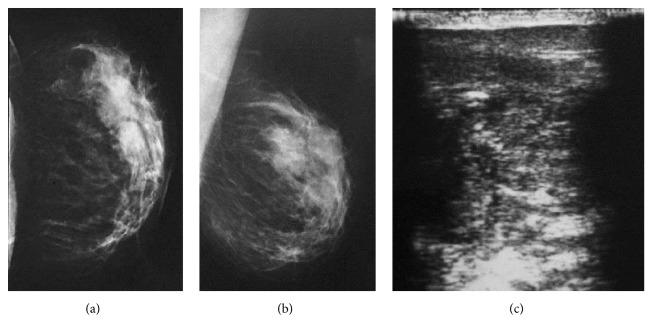
Mammogram images (a and b) showing a mass with irregular posterior limits, which on ultrasound image (c) is a hypoechoic mass with irregular and indistinct margins at the 2 o'clock position of the left breast measuring approximately 4.2 cm in maximal diameter* (Collection of the Department of Radiology, University Teaching Hospital of Lomé)*.

A 42-year-old nonlactating and nonpregnant woman presented with a painful palpable mass in her left breast for three months. She was initially self-treated with antibiotic in automedication with regression of pain, but the mass persists. One month after pains recurred with a cutaneous fistula of which pus bacteriologic analysis isolated* Staphylococcus aureus *sensible to penicillin. She underwent ultrasound and mammogram and an ultrasound-guided core needle biopsy of the mass. The mammogram demonstrated a bilobar mass of upper and external region. The masse had irregular posterior limits and was not associated with focal architectural distortion or cluster of microcalcifications (Figures 2(a) and 2(b)). Ultrasound revealed a hypoechoic mass with irregular and indistinct margins at 2 o'clock position of the left breast measuring approximately 4.2 cm in maximal diameter (Figure 2(c)). Color Doppler detected peripheral vascularity of the mass. There were multiple enlarged left axillary lymph nodes detected on ultrasound. The mass was classified as highly suggestive of malignancy according to the American College of Radiology Breast Imaging Reporting and Data System (ACR BI-RADS: 5). Histopathologic exam of the biopsy specimen showed an invasive canalar carcinoma with subacute mastitis.

### 2.3. Case 3

**Figure 3:**
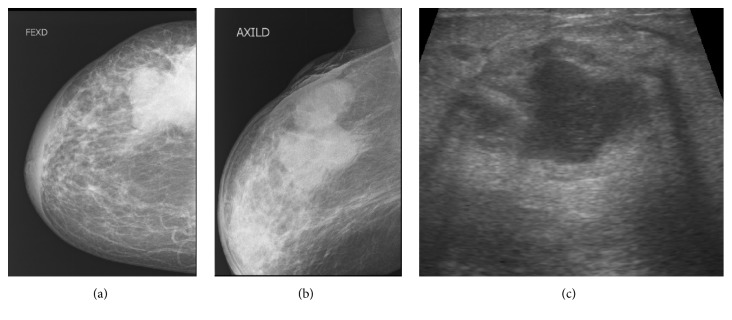
Mammogram images (a and b) presenting a mass with polylobed contours, associated with a small cluster of microcalcifications at 11 o'clock position of the right breast, with a thickening of the retroareolar skin facing the masse, which was confirmed as a hypoechoic mass with irregular and indistinct margins measuring approximately 4.6 cm in maximal diameter on ultrasound exam (c)* (Collection of the Department of Radiology, University Teaching Hospital of Lomé)*.

A 63-year-old menopaused woman was referred to a clinic for right breast recidivist abscess for two mouths. At the exam, there is a fistula in 11 o'clock position the right breast from which came out pus, associated with a palpable mass. She was initially treated by ultrasound-guide drainage of the abscess, associated with a correct antibiotic correlated with the sensitivity of the germ* (staphylococcus aureus)* isolated by bacteriology at the clinic without any improvement. At the hospital, she underwent ultrasound and mammogram. The mammogram revealed a mass with polylobed contours, associated with a small cluster of microcalcifications at 11 o'clock position of the right breast, with a thickening of the retroareolar skin (Figures 3(a) and 3(b)).

Ultrasound confirmed a hypoechoic mass with irregular and indistinct margins at the same position as mammogram measuring approximately 4.6 cm in maximal diameter, with thickening of the skin facing the mass and the retroareolar region (Figure 3(c)).

The mass was classified as highly suggestive of malignancy according to the American College of Radiology Breast Imaging Reporting and Data System (ACR BI-RADS: 5). The recommended ultrasound-guided core needle biopsy of the left breast mass was done. Histopathologic exam confirms an infiltrative canalar carcinoma with subacute mastitis.

## 3. Discussion

The association of breast cancer and abscess is known, but this malignant inflammatory disease is rare in daily practice. Its incidence is increasing since 1990 [2, 3]. Beyrouti et al. [4] reported on their series in Tunisia 02 cases of malignant mastitis abscessed on 104 cases of pyogenic breast abscesses collected in 14 years. Zaki et al. [5] found 03 cases out of 100 cases collected over a period of 17 years in Niger in 2015. Trop et al. [6] in a review of 20 studies that included 975 cases of breast abscess, 6 cases (0.6%) of inflammatory carcinoma were encountered.

### 3.1. Ultrasound

The breast abscess aspect in ultrasound is a very hypoechoic oval or circular mass, with sharp or regular contours and less or good acoustic transmission [7, 8]. There is no real ultrasound specific sign to evoke cancer, but ultrasound is the method of choice for controlling the therapeutic response. This response is crucial because when there is no improvement; a biopsy should be done necessary.

### 3.2. Mammography

The abscess can present different aspects in mammography. Most of the time, it is a circular mass with slightly irregular contours; its limits are typically unclear and blurry, due to the peripheral edema; the extension of the edema in the retroareolar region is considered as a typical sign, but it is not always present. In a very dense tissue, it can be represented by a very discreet increase of density, nonspecific or sometimes scarcely perceptible. Cutaneous edema is presented as a crosslinking and thickening of the skin; sometimes gaseous inclusions or a hydroaerial image are observed [9].

### 3.3. Biopsy

In all inflammatory breast processes, the proceeding depends on the response to antibiotics. When there is no improvement after a well-conducted antibiotic treatment and there is a real suspicion of cancer, ultrasound-guided core needle biopsy or fine needle aspiration cytology is necessary [10]. There is no mammographic standard to surely exclude a risk of cancer.

### 3.4. Bacteriology

The* Staphylococcus aureus* was the germ the most frequently met [4], even in nonlactating and nonpuerperal breast abscess [11, 12]. Numerous histological studies find infiltrative canalar carcinoma of breast associated with abscess [13, 14]. But none of them thoroughly investigated the association of these two pathologies.

### 3.5. Histopathology

Several cases of cancers presenting as an abscess have been described, mostly outside the breastfeeding period. These are most often cases of pure primary squamous cell carcinoma of the breast [15–18] but also primary lymphoma [18, 19] or even lymphoepithelioma-like carcinoma originally presented as an abscess, although it is very rare [20]. Even in breastfeeding an abscess may turn out to be cancer [21]. A real breast cancer can hide behind a typical breast abscess.

In our cases, histological examination had found only infiltrating ductal carcinomas associated with fibroinflammatory stroma. We consider that this aspect, in our medical context characterized by a delay in consultation and traditional medicine practices with infectious risks, such as potions or breast scarification, was an infection by pyogenic germs of the breast on an existing cancer which has not been treated in time.

## 4. Conclusion

The nonpuerperal abscesses can pose a differential diagnosis problem with the inflammatory cancers. The rate of associated malignancies with breast abscess is low. The percutaneous ultrasonography-guided drainage must be proposed in first intention in association with antibiotics to treat the abscesses of the breast, with ultrasound control at the end of the treatment. In case of recidivism of failure an ultrasound-guided biopsy is indicated in order to make histological confirmation.
